# Sensors for Smoking Detection in Epidemiological Research: Scoping Review

**DOI:** 10.2196/52383

**Published:** 2024-10-30

**Authors:** Giuliana Favara, Martina Barchitta, Andrea Maugeri, Roberta Magnano San Lio, Antonella Agodi

**Affiliations:** 1 Department of Medical and Surgical Sciences and Advanced Technologies “GF Ingrassia” University of Catania Catania Italy

**Keywords:** smoking, tobacco smoke, smoke exposure, cigarette smoking, wearable sensor, public health

## Abstract

**Background:**

The use of wearable sensors is being explored as a challenging way to accurately identify smoking behaviors by measuring physiological and environmental factors in real-life settings. Although they hold potential benefits for aiding smoking cessation, no single wearable device currently achieves high accuracy in detecting smoking events. Furthermore, it is crucial to emphasize that this area of study is dynamic and requires ongoing updates.

**Objective:**

This scoping review aims to map the scientific literature for identifying the main sensors developed or used for tobacco smoke detection, with a specific focus on wearable sensors, as well as describe their key features and categorize them by type.

**Methods:**

According to the PRISMA-ScR (Preferred Reporting Items for Systematic Reviews and Meta-Analyses extension for Scoping Reviews) protocol, an electronic search was conducted on the PubMed, MEDLINE, and Web of Science databases, using the following keywords: (“biosensors” OR “biosensor” OR “sensors” OR “sensor” OR “wearable”) AND (“smoking” OR “smoke”).

**Results:**

Among a total of 37 studies included in this scoping review published between 2012 and March 2024, 16 described sensors based on wearable bands, 15 described multisensory systems, and 6 described other strategies to detect tobacco smoke exposure. Included studies provided details about the design or application of wearable sensors based on an elastic band to detect different aspects of tobacco smoke exposure (eg, arm, wrist, and finger movements, and lighting events). Some studies proposed a system composed of different sensor modalities (eg, Personal Automatic Cigarette Tracker [PACT], PACT 2.0, and AutoSense).

**Conclusions:**

Our scoping review has revealed both the obstacles and opportunities linked to wearable devices, offering valuable insights for future research initiatives. Tackling the recognized challenges and delving into potential avenues for enhancement could elevate wearable devices into even more effective tools for aiding smoking cessation. In this context, continuous research is essential to fine-tune and optimize these devices, guaranteeing their practicality and reliability in real-world applications.

## Introduction

According to the World Health Organization, tobacco smoke poses a major public health issue, causing approximately 8 million deaths annually worldwide [1]. It is a prominent contributor to noncommunicable diseases, such as cardiovascular diseases, chronic respiratory diseases, cancer, and diabetes, accounting for 1 out of every 6 deaths caused by these diseases [2,3]. In line with the sustainable development goals, reducing tobacco use is an essential requirement to achieve progress in the prevention and control of noncommunicable diseases, as well as for monitoring tobacco control efforts [4]. However, smoking cessation is often hindered by low awareness of the health risks related to tobacco smoke [2]. In order to tackle this problem, there are multiple strategies available to aid individuals in their journey to quit smoking, and it is crucial to establish regular data collection on smoking habits and individual exposures [5,6].

A number of conventional approaches (eg, portable puff instruments, self-report questionnaires, and ecological momentary assessments) have been employed to monitor smoking habits in epidemiological research [2]. However, these methods have shown limited efficacy in accurately detecting smoke exposures, due to memory biases and underreporting by individuals. For these reasons, they do not offer valuable tools to support effective public health interventions [7]. More recently, wearable sensors have emerged as a potential approach for detecting smoking exposure in individuals. Broadly speaking, these sensors are usually intended to be worn on the body and measure various physiological or environmental parameters and behaviors of smoke exposure. It is intriguing that these devices comprise a blend of distinct sensor modalities (such as electrical, inertial, and acoustic) and a system of multiple sensors [8,9]. However, it is important to note that this field of research is still evolving, indicating that there is currently no single wearable device that exhibits high accuracy in detecting smoking events in all situations, isolating puffs and smoke inhalations, or evaluating smoke exposure.

In this context, the systematic review conducted by Imtiaz et al [2] provides a summary of recent innovative approaches (ie, individual and multisensor combinations, various body locations, and signal processing methodologies) of cutting-edge wearable sensors designed for monitoring cigarette smoking in real-world conditions, including studies published from 1990 to 2019. However, the following years have been marked by the COVID-19 pandemic, a globally impactful event that may have influenced the interest and potential applications of new technologies for monitoring lifestyles. Additionally, given that sensor use is a rapidly evolving field, it is necessary to consistently provide updates in the scientific literature.

Considering this, our scoping review was undertaken to offer an updated summary of studies published until March 2024, presenting the application of wearable sensors for monitoring cigarette smoking and smoke exposure, and focusing on both single-sensor and multisensor approaches.

## Methods

The review methodology was employed by following these steps: (1) identifying the research question (RQ); (2) defining the search strategy and protocol; (3) conducting a literature search; and (4) collecting data from the included studies.

Thus, the RQ of our systematic review was as follows: what is the state of the art regarding the development and use of sensors (particularly wearable sensors) for monitoring smoking habits in epidemiological studies? Additionally, what are the main types of sensors and how do they differ in terms of modalities and potential applications?

This scoping review was conducted according to the PRISMA-ScR (Preferred Reporting Items for Systematic Reviews and Meta-Analyses extension for Scoping Reviews) protocol (Multimedia Appendix 1) [10]. Two authors (GF and AM) independently conducted a literature search and selected potentially relevant articles from inception to March 10, 2024, using the PubMed, MEDLINE, and Web of Science databases. The electronic search strategy consisted of the following keywords: (“biosensors” OR “biosensor” OR “sensors” OR “sensor” OR “wearable”) AND (“smoking” OR “smoke”).

Articles were included in the scoping review if they reported studies describing, developing, or applying sensors for the detection of tobacco smoke exposure, with the potential to be wearable. Thus, articles were included if they met the following criteria: (1) were written in English; (2) involved human participants; (3) described, developed, or applied sensors that could be wearable; (4) analyzed active or passive exposure to tobacco smoke; (5) were conducted in a laboratory or the community; and (6) described protocols according to the RQ of the scoping review. By contrast, articles were excluded if they met the following criteria: (1) did not fit into the RQ of the scoping review; (2) reported studies on sensors that were not or potentially not wearable; (3) did not analyze active or passive exposure to tobacco smoke; (4) presented only the biochemical or biomolecular applications of sensors; and (5) were letters, comments, abstracts, editorials, reviews, systematic reviews, or meta-analyses.

By using a data abstraction form designed for this scoping review, 2 authors (GF and AM) collected the following information from all the included studies: article characteristics (eg, first author, publication year, and country of origin), study characteristics (eg, study design), sensor specifications (eg, type of wearable sensors, parameters that were measured, accuracy, data availability, and autonomous or integrated system), and applications proposed.

Diverse opinions between investigators were resolved through discussion between the 2 authors or by consulting a third author (AA). The studies were categorized based on the types of wearable devices employed, with particular attention given to distinguishing between single-device applications and combinations or systems of these devices.

## Results

### Study Selection

**Figure 1:**
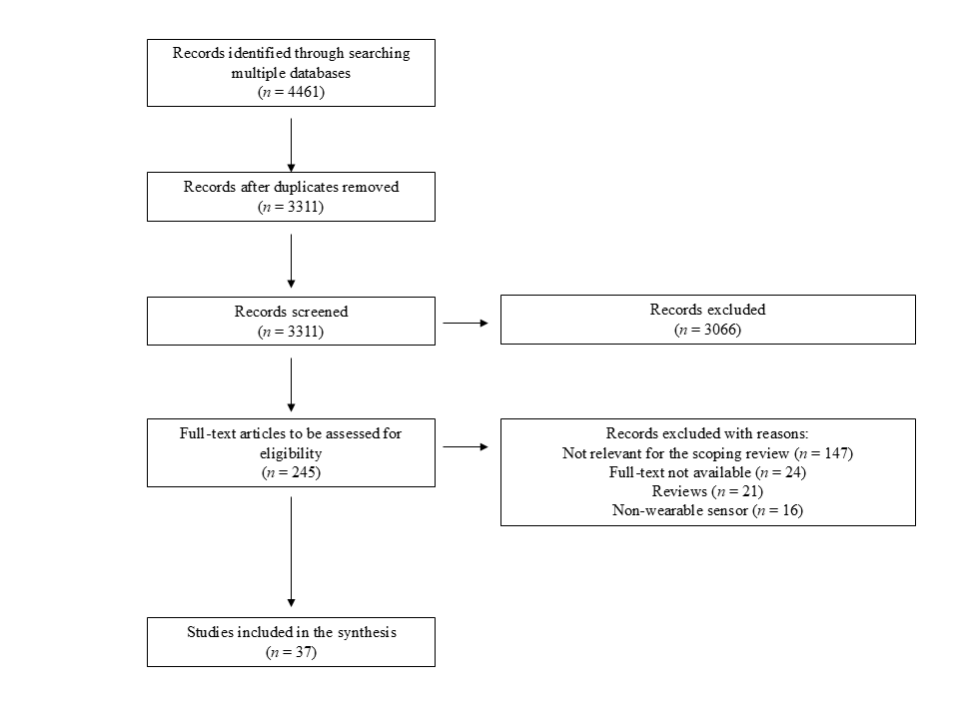
Flowchart illustrating the selection of studies included in the scoping review.

Following the removal of duplicates, a total of 3311 articles were initially identified through the literature search. Through the screening process of titles and abstracts, 3066 articles were excluded, leaving 245 full-text articles that were thoroughly evaluated for eligibility. Among these, 208 were excluded for the following reasons: 147 were not relevant for the objective of the current scoping review, 24 were without full text, 21 were reviews, and 16 used nonwearable sensors. Thus, the remaining 37 studies were considered eligible and were included in this scoping review (Figure 1).

### Characteristics of the Studies Included

The vast majority of studies included in this scoping review originated from the United States (n=27). In particular, 16 studies described single wearable devices or devices in combination, 15 studies described multisensor systems, and 6 studies described other innovative strategies for the detection of cigarette smoke exposure. All of the studies included in this review were conducted within the general population, with a particular emphasis on individuals who smoke.

In the following sections, we grouped the studies included by the type of wearable sensor considered, as follows: (1) a single elastic band or a combination of elastic bands; (2) multisensor systems (ie, “Personal Automatic Cigarette Tracker [PACT],” “PACT 2.0,” and “AutoSense”); and (3) other alternative strategies.

### Wearable Sensors Based on Elastic Bands

Sixteen studies focused on detailing the design or application of wearable sensors [8,11-14] or a combination thereof [15-23] to detect different aspects of tobacco smoke exposure (Table 1). While numerous studies commonly suggested sensors mounted in fixed locations, these approaches did not permit a comprehensive determination of tobacco smoke exposure among specific subgroups of individuals [24]. In this context, tobacco test strips are among the commercially available options for assessing nicotine levels in urine and saliva samples [25].

**Table 1:** Summary of studies exploring wearable sensors based on elastic bands.

Study	Country	Type of wearable	Application	Sensor modality	Accuracy	Data availability	Autonomous/integrated
Cole et al [26], 2021	United States	Smartwatch and smartphone app	To validate a smartwatch for the examination of smoking temporal patterns	Interpuff intervals and puff duration	—^a^	User and researcher	Integrated with a smartphone app
Dar [19], 2018	Israel	Smartwatch and wristbands	To investigate whether smoker monitoring through the SmokeBeat app would result in a reduction in smoking	Hand-to-mouth gestures	—	User and researcher	Integrated with a smartphone app
Horvath et al [20], 2021	United States	Smartband and smartphone	To propose a protocol for aiding daily smokers in their attempt to quit smoking	Hand-to-mouth gestures	—	User and researcher	Integrated with a smartphone app
Joyce et al [21], 2021	United States	Smartwatch or smartband	To assess the viability, acceptability, and efficacy of a smoking cessation program utilizing smartwatch technology and incentive-based strategies	Hand-to-mouth gestures	—	User and researcher	Integrated with a smartphone app
Lopez-Meyer et al [18], 2013	United States	Wristband	To describe a sensor for monitoring gestures in cigarette smokers	Hand-to-mouth gestures	Sensitivity: 90%	User and researcher	Data logger unit
Maguire et al [15], 2022	United States	Smartwatch and finger elastic band	To develop a smoking detection system to classify presmoking behaviors	Arm, wrist, and finger movements	Accuracy: 80.6%	User and researcher	Integrated with a smartphone app
Morriscey et al [22], 2018	United States	Smartwatch and smartphone app	To determine the sensitivity and specificity of the smartphone app	Hand-to-mouth gestures	Sensitivity: 22.5%-41.7%	User and researcher	Integrated with a smartphone app
Parate et al [12], 2014	United States	Wristband	To design a mobile solution to capture changes in the orientation of a person’s arm	Hand-to-mouth gestures	Accuracy: 95.7%; precision: 91%; recall: 81%	User and researcher	Integrated with a smartphone app
Quintana et al [27], 2018	United States	Silicone wristband	To assess the effectiveness of silicone wristbands as personal nicotine samplers	Nicotine level	—	Researcher	Autonomous
Raiff et al [13], 2014	United States	Wrist, elbow, and shoulder bands	To develop and test the ability of inertial sensors to detect cigarette smoking	Arm movements	—	User and researcher	Integrated with a tablet
Schnall et al [23], 2022	United States	Smartwatch and smartphone app	To assess the feasibility and efficacy of the Lumme Quit Smoking mobile app	Hand-to-mouth gestures	—	User and researcher	Integrated with a smartphone app
Senyurek et al [14], 2019	United States	Chest band	To develop and test a machine learning model for smoking recognition	Breathing signals	Accuracy: 80.0%	—	—
Skinner et al [17], 2018	United Kingdom	Smartwatch	To present a system for passive detection of cigarette smoking	Hand-to-mouth gestures	—	—	Integrated with a smartphone
Tai et al [11], 2020	United States	Forearm band	To detect nicotine levels in the sweat of subjects inhaling cigarette smoke	Nicotine level	—	Researcher	Integrate with a computer
Takur et al [8], 2022	India	Wristband	To develop a modeling framework for smoking recognition	Hand-to-mouth gestures	Accuracy: 98.7%	User and researcher	Data logger unit integrated with a computer
Zhai et al [16], 2020	Belgium	A system that includes: an electric lighter, wristband, and chest patch	To reveal temporal patterns of smoking behaviors	Hearth rate, breathing, and hand-to-mouth gestures	—	User and researcher	Integrated with a smartphone app

^a^Not applicable.

Nonetheless, achieving a personalized and real-time assessment of individual exposure remains a challenge, emphasizing the need for further efforts to develop tailored preventive strategies accordingly. In an effort to address this concern, some authors presented a strategy to bridge the gap between fixed tobacco sensors and nicotine test strips. In particular, they proposed a noninvasive and wearable forearm band designed for continuous and real-time monitoring of nicotine levels in human sweat after nicotine inhalation. The device comprised a flexible electrode array connected to an electronic circuit, demonstrating favorable sensitivity and stability in both smokers and nonsmokers alike [11].

A recent study introduced a smoking cessation system aimed at predicting presmoking movements, such as reaching for a pack of cigarettes or lighting a cigarette, which are associated with smoking behaviors. This system utilizes an accelerometer embedded in a smartwatch to capture arm and wrist movements, along with a wearable finger sensor to measure the bending angle of the user’s index finger. Their results showed that a model integrating data from both the smartwatch and finger sensor achieved higher accuracy in classifying presmoking activities compared to the model relying solely on the smartwatch. These findings lay the groundwork for developing an effective smoking cessation strategy that utilizes the combined input from these devices [15].

The authors who designed the “RisQ” mobile solution made additional endeavors to differentiate smoking episodes by validating a solution based on a wristband capable of detecting arm and wrist movements during smoking, as well as hand-to-mouth gestures, utilizing a machine learning model. Specifically, the authors compared the performance of 2 different algorithms in accurately identifying hand-to-mouth gestures, puffs, and smoking events. The authors proposed a pipeline aimed at effectively and promptly detecting smoking gestures in real time, yielding favorable accuracy, precision, and recall values [12]. In this context, a study conducted in 2020 assessed recurring patterns of smoking behaviors in real-life settings. The authors proposed a system comprising a wristband (capturing arm movements and skin impedance data), a chest patch (gathering electrocardiogram [ECG] information), and an electric lighter (detecting smoking events) to collect data regarding the contextual aspects in which individuals smoke. Notably, the study revealed intriguing differences in temporal patterns, encompassing weekly, daily, and time-of-day variations, as well as variations in emotional states during smoking episodes [16].

Some researchers introduced an innovative use of wearable devices for the real-time detection of smoking activity. They developed a wearable wristband that integrated 6-axial inertial sensors to gather data on physical activities, such as walking, running, walking upstairs, and walking downstairs, as well as smoking events. Additionally, they leveraged the collected data to create a machine learning model capable of distinguishing smoking activity from various daily activities, demonstrating promising performance [8]. Similarly, another study aimed to assess the capability of 4 inertial sensors (positioned on the wrist, elbow, and arm) to capture arm movements. Interestingly, the authors compared 2 distinct algorithms, namely support vector machine (SVM) and edge-detection–based learning. The results highlighted that the SVM model had superior performance in recognizing smoking events and interpuff intervals [13].

Similarly, a study published in 2019 compared the performance of various machine learning models with a focus on detecting smoke inhalations, utilizing a wearable chest band to capture breathing signals. The findings indicated that novel deep learning approaches may offer a more accurate method for detecting smoke inhalations than conventional machine learning models [14]. A validation study presented a system designed for the detection of passive cigarette smoking. The system consisted of a smartwatch equipped with a combination of accelerometers and gyroscope sensors, enabling the detection of hand movements associated with cigarette smoking [17]. Similarly, some authors validated a wristband that transmitted hand-to-mouth gestures to a receiver positioned on the user’s chest. Interestingly, the proposed sensor exhibited good sensitivity and provided a methodology to differentiate hand-to-mouth gestures originating from smokers [18].

Although mobile apps for smoking cessation are becoming more widely accessible, their effectiveness is yet to be proven. In this context, a pilot trial being carried out in the United States is evaluating the potential of utilizing a smartband and smartphone for real-time monitoring, detecting smoking, and delivering concise mindfulness interventions to diminish smoking. The trial protocol seeks to provide insights into the practicability of employing the combined use of a smartband and smartphone [20]. An additional pilot study investigated SmokeBeat, an inventive app tailored for use with smartwatches and wristbands. This app processes information and utilizes embedded sensors in wearables to identify hand-to-mouth gestures in real time. The study’s authors highlighted that the SmokeBeat algorithm accurately identified more than 80% of smoking episodes, and participants in the experimental group demonstrated a notable decrease in smoking rates throughout the 30-day trial [19]. SmokeBeat was also employed to assess the feasibility, acceptability, and effectiveness of a smoking cessation program designed for low-income pregnant smokers, utilizing smartwatch technology and incentive-based strategies. Reductions in smoking were noted in both the control and intervention groups across all pilot studies. While the utilization of the SmokeBeat program did not lead to a significant improvement in cessation rates, its feasibility and acceptability were deemed moderately high [21]. Similarly, another study demonstrated SmokeBeat’s good sensitivity and specificity in cigarette detection following a learning period [22]. Remarkably, a pilot study indicated outstanding feasibility and acceptability in utilizing the “Lumme Quit Smoking” mobile app in conjunction with a smartwatch, aiming to enhance smoking cessation outcomes among individuals with HIV [23].

In this context, a pilot feasibility study was recently conducted to study temporal patterns and characteristics of smoking among adult smokers in a controlled laboratory setting. The authors compared the agreement of recording smoking characteristics by comparing the Polar M600 smartwatch that recorded accelerometer data to identify the durations of puff and interpuff intervals using Automated Smoking Perception and Recording (ASPIRE) software and the pocket Clinical Research Support System (CReSS) topography device that uses video observation. The results suggested that the ASPIRE approach is more accurate than the CReSS method for passively monitoring smoking behavior. Moreover, the ASPIRE approach was more accurate than the CReSS method for measuring puff and interpuff intervals. In fact, the ASPIRE approach consistently produced a higher count of puffs and maintained more stable durations of interpuff intervals in comparison to the CReSS method, aligning both methods with the visually observed puff count. After filtering out implausible data from the CReSS method, both the ASPIRE approach and CReSS method provided consistent results for both puff duration and interpuff intervals [26]. A different study has proposed that uncomplicated silicone wristbands could serve as potential passive samplers for monitoring tobacco product exposure in children. Additionally, they have emerged as a noteworthy strategy for epidemiological and intervention studies. The researchers investigated the efficacy of 2 silicone wristbands for sampling personal nicotine levels in children, which were worn for durations of 7 days and 2 days, respectively. To achieve this, they compared the nicotine levels detected by the wristbands to urinary cotinine, a nicotine metabolite, measured in the urine of children exposed to contaminants in tobacco smoke or vapor from electronic cigarettes, as well as those living in nonsmoking households. The nicotine detected in the wristbands worn for 2 days exhibited a high correlation with urinary cotinine concentration, mirroring the correlation found in wristbands worn for 7 days. Moreover, the nicotine amounts recorded in the wristbands for both 2 and 7 days were significantly correlated [27].

### Multisensor System: PACT

Five studies used the PACT to monitor tobacco smoke exposure (Table 2). The PACT is a comprehensive system composed of various sensor modalities [2,28-31]. Its primary objective is to track smoking episodes by detecting hand-to-mouth gestures that precede smoke inhalations. The PACT system comprises several components, including a chest module, a wrist or forearm band, an instrumented lighter, and a data logger. Specifically, the PACT system consists of the following elements. First, wearable respiratory inductance plethysmograph (RIP) sensors that are mounted in abdominal and thoracic bands. These sensors capture changes in breath volume, which are indicative of the expansion and contraction of the subject’s lungs. Second, a radio frequency (RF) proximity sensor that is used to detect hand-to-mouth gestures. It consists of a transmitter positioned on the wrist and a receiver positioned on the chest. This sensor records the proximity of the hand to the mouth during smoking episodes. Third, a portable data logger that captures and stores the signals from the RIP sensors and RF proximity sensors. Additionally, the PACT system includes a self-report button that allows users to manually register each smoking event [2,32]. This feature enables users to input and record their smoking activities within the PACT system, providing an additional means of tracking and monitoring their smoking behavior [2,32].

**Table 2:** Studies exploring the Personal Automatic Cigarette Tracker system.

Study	Country	Type of wearable	Application	Sensor modality	Accuracy	Data availability	Autonomous/integrated
Lopez-Meyer et al [29], 2013	United States	Personal Automatic Cigarette Tracker (PACT) system that includes: thoracic, abdominal, and wrist bands; a chest receiver; a portable plethysmograph; and a data logger	To describe the development of a noninvasive wearable sensor system	Hand-to-mouth gestures and breathing	Precision: 87%; recall: 80%	Researcher	Autonomous data logger unit
Lopez-Meyer et al [30], 2012	United States	PACT system that includes: thoracic, abdominal, and wrist bands; a chest receiver; a portable plethysmograph; and a data logger	To apply a machine learning model for identifying cigarette smoke inhalations from wearable sensor data	Hand-to-mouth gestures and breathing	Precision: >87%; recall: >80%	Researcher	Autonomous data logger unit
Patil et al [28], 2013	United States	PACT system that includes: thoracic, abdominal, and wrist bands; a chest receiver; a portable plethysmograph; and a data logger	To apply a machine learning model for detecting cigarette smoke inhalations from respiratory signals	Hand-to-mouth gestures and breathing	Accuracy: 80%	Researcher	Autonomous data logger unit
Patil et al [32], 2014	United States	PACT system that includes: thoracic, abdominal, and wrist bands; a chest receiver; a portable plethysmograph; and a data logger	To analyze the factors affecting the output quality of the abdominal and thoracic bands	Hand-to-mouth gestures and breathing	F-score: 94%	Researcher	Autonomous data logger unit
Sazonov et al [31], 2013	United States	PACT system that includes: thoracic, abdominal, and wrist bands; an airflow sensor; a chest receiver; a portable plethysmograph; and a data logger	To describe the prototype of the sensor system and preliminary results of initial testing	Hand-to-mouth gestures, breathing, and oral and nasal airflow	—^a^	Researcher	Autonomous data logger unit

^a^Not applicable.

A validation study found that the accuracy of the classifier depends on the signals recorded, with abdominal breathing and hand gestures playing a significant role in detecting smoke inhalations. Additionally, the authors evaluated the impact of anthropometric measures on the quality of data captured by the PACT system. They suggested that the BMI and posture of individuals may influence the quality of smoking breathing signals [32]. Furthermore, another study demonstrated the feasibility of detecting smoke inhalation using the PACT system, indicating that each individual has unique characteristics in their response during smoking [28].

In the context of the PACT system, a study demonstrating the feasibility of automatically recognizing smoke inhalations was conducted. By applying the SVM algorithm, the authors found that breathing patterns exhibited individual characteristics. Subject-dependent models showed higher precision and recall values compared to subject-independent models [29]. In a further study, the same authors reported the findings of a subject-independent model for detecting smoke inhalations using data collected through the PACT system. In particular, the SVM model achieved high precision and recall values for detecting cigarette smoke inhalations [30].

A laboratory study proposed an intriguing application of the PACT system for reliable monitoring of smoking episodes in real-life settings. The findings revealed that smoking breathing patterns exhibit individual characteristics and display a strong correlation with hand-to-mouth gestures. As a result, the authors suggested that the PACT system can be employed to automatically assess daily smoking habits and evaluate the efficacy of behavioral and pharmacological interventions [31].

### Multisensor System: PACT 2.0

Some studies (n=4) introduced the PACT system version 2.0, an enhanced iteration designed for automated real-time monitoring of smoking behavior (Table 3) [33-36]. Interestingly, the PACT 2.0 system eliminates the need for manual input from users as all smoking events are automatically detected and recorded by the sensors. In addition to capturing smoking data, the PACT 2.0 system collects supplementary information on smoking behaviors, including GPS location data and ECG data. It further offers users real-time feedback on their smoking behavior through a smartphone interface. This advanced system comprises a combination of components, including a chest module, a wrist or forearm module, an instrumented lighter, and a dedicated smartphone app.

**Table 3:** Studies exploring the Personal Automatic Cigarette Tracker 2.0 system.

Study	Country	Type of wearable	Application	Sensor modality	Accuracy	Data availability	Autonomous/integrated
Imtiaz et al [33], 2017	Switzerland	Personal Automatic Cigarette Tracker (PACT) 2.0 system that includes: a wristband, chest band, and smart lighter	To describe and validate a multisensory wearable system for monitoring cigarette smoking behavior	Hand-to-mouth gestures, breathing, lighting events, and heart rate	—^a^	Researcher	Autonomous data logger unit
Imtaiz et al [34], 2019	United States	PACT 2.0 system that includes: 2 cables and adhesive electrodes	To describe a novel method to identify smoking events	Heart rate, breathing, and hand-to-mouth gestures	Sensitivity: 87%; F-score: 79%	Researcher	Autonomous data logger unit
Senyurek et al [35], 2019	United States	PACT 2.0 system that includes: a wristband and smart lighter	To develop a robust sensor-based monitoring solution to detect smoking events	Hand-to-mouth gestures and lighting events	Accuracy: 84%; F-score: 91%	User and researcher	Integrated with a smartphone app
Senyurek et al [36], 2019	United States	PACT 2.0 system that includes: a wristband and smart lighter	To describe a novel method to identify smoking events	Hand-to-mouth gestures and lighting events	Accuracy: 84%; F-score: 91%	User and researcher	Integrated with a smartphone app

^a^Not applicable.

The chest module of the PACT 2.0 system captures various types of data, including breathing patterns recorded by inductive and bioimpedance respiratory sensors, cardiac activity measured by an ECG sensor, chest movement monitored by a 3-axis accelerometer, hand-to-mouth proximity detected by an RF receiver, and geospatial information obtained through a GPS receiver. On the other hand, the hand module records hand-to-mouth gestures using an inertial measurement unit (IMU) integrated with an RF transmitter. Additionally, the hand module serves as a pedometer, measuring the user’s steps. The instrumented lighter within the PACT system is capable of detecting when the user is lighting a cigarette, as well as monitoring the smoking process and detecting when the cigarette is extinguished. To further enhance the user experience, the system is accompanied by a smartphone app that offers real-time feedback on smoking behavior. This includes information, such as the number of cigarettes smoked, the timing of each smoking event, and the associated location [2].

In a study conducted on a sample of 40 smokers in real-life settings, the PACT 2.0 system was developed and validated for monitoring cigarette smoking. The system demonstrated high acceptability and reliability, serving as an effective platform for detecting smoking behaviors [33]. In another investigation, the authors put forth changes in heart rate, along with breathing signals and body motion, as specific indicators of cigarette smoking, by employing the chest module of the PACT 2.0 system for this purpose. Utilizing an SVM model, they successfully developed an automated detection system for smoking events, achieving a high level of accuracy [34].

In another study, a combination of an instrumented lighter and a wrist IMU from the PACT 2.0 system was used in a group of smokers. The results indicated that integrating the IMU and instrumented lighter holds potential for studying smoking behavior in natural settings, resulting in higher accuracy values for the SVM classifier [35]. In a subsequent study, the temporal regularity of hand gestures was identified as a novel approach for detecting smoking events using the wrist IMU and PACT 2.0 lighter. Interestingly, this study revealed a high level of regularity in hand-to-mouth gestures during smoking episodes [36].

### Multisensor System: AutoSense

In recent advancements, there have been proposals to leverage multiple wearable sensors in order to enhance accuracy, simplify signal detection, and combine various sensor modalities. Notably, 6 studies have put forth the utilization of AutoSense technology to monitor cigarette exposure and offer real-time feedback to individuals (Table 4) [37-42]. This technology employs a combination of wearable sensors that capture physiological signals associated with cardiovascular, respiratory, and thermoregulatory activities.

**Table 4:** Studies exploring the AutoSense system.

Study	Country	Type of wearable	Application	Sensor modality	Accuracy	Data availability	Autonomous/integrated
Battalio et al [39], 2021	United States	AutoSense system that includes: a chest band, wristbands, and electrocardiogram (ECG) electrodes	To investigate whether the delivery of a prompt could perform stress management and smoking behaviors	Hearth rate, breathing, and hand-to-mouth gestures	—^a^	User and researcher	Integrated with a smartphone
Chatterjee et al [41], 2016	United States	AutoSense system that includes: a chest band, wristbands, and ECG electrodes	To estimate cigarette craving during smoking abstinence	Hearth rate, breathing, and hand-to-mouth gestures	—	User and researcher	Integrated with a smartphone
Chatterjee et al [40], 2020	United States	AutoSense system that includes: a chest band, wristbands, and ECG electrodes	To automatically detect smoking “opportunity context”	Hearth rate, breathing, and hand-to-mouth gestures	—	User and researcher	Integrated with a smartphone
Hernandez et al [38], 2021	United States	AutoSense system that includes: a chest band, wristbands, and ECG electrodes	To deliver mindfulness-based strategies in real-time among individuals attempting to quit smoking	Hearth rate, breathing, and hand-to-mouth gestures	—	User and researcher	Integrated with a smartphone
Nakajima et al [37], 2020	United States	AutoSense system that includes: a chest band, wristbands, and ECG electrodes	To examine relationships between stress and smoking behavior and lapse among smokers motivated to quit smoking	Hearth rate, breathing, and hand-to-mouth gestures	Sensitivity: 80%	User	Integrated with a smartphone
Saleheen et al [42], 2015	United States	AutoSense system that includes: a chest band, wristbands, and ECG electrodes	To propose and evaluate a new model for detecting a smoking lapse	Hearth rate, breathing, and hand-to-mouth gestures	—	User and researcher	Integrated with a smartphone

^a^Not applicable.

AutoSense integrates various wearable sensors, including (1) a chest band equipped with a RIF sensor to capture respiration patterns and lung volume; (2) two chest ECG electrodes to measure electrical heart activity; and (3) inertial sensors placed on each wrist to detect movement patterns, hand-to-mouth gestures, and changes in body posture. All sensors continuously transmitted the collected data to a mobile phone for analysis and monitoring [2,42].

In a noteworthy study, the use of AutoSense technology was explored to identify stress states (cStress) and to predict first lapse smoking episodes (puffMarker) during a clinical study of smoking cessation. These findings suggested that heart rate and cStress could serve as useful predictors of smoking lapse [37]. Furthermore, this scoping review included a protocol that focused on recruiting motivated individuals who wanted to quit smoking. The protocol outlined a microrandomized controlled trial that would utilize the AutoSense system to deliver mindfulness strategies during the quit smoking attempt [38]. Another trial conducted on 75 smokers who expressed a desire to quit smoking and wore the AutoSense system, provided valuable insights for enhancing just-in-time stress management interventions aimed at preventing smoking relapse [39].

In this scenario, another notable study used data collected by the AutoSense system to identify common contexts of smoking “opportunity” (eg, smoking lapse, overeating or binge drinking, etc), which can either discourage or encourage individuals from engaging in adverse daily-life behaviors [40]. In a related study, the AutoSense system was employed to investigate the phenomenon of cigarette craving during smoking abstinence. Interestingly, individuals who reported high cravings experienced higher levels of stress during the hours of the day when the craving was elevated, compared to those with low cravings [41].

Moreover, a research group proposed the application of the AutoSense system in a population of abstinent smokers to detect the occurrence of a first lapse. By using an SVM classifier, the authors demonstrated the ability to detect the timing of the first lapse in smoking cessation within real-life settings [42].

### Other Strategies

In our scoping review, we identified 6 studies that proposed alternative sensor modalities for the detection of smoking behavior (Table 5). One intriguing approach involves leveraging the distinct acoustic characteristics of smoking breaths, which differ from those of nonsmoking breaths. This opens up the potential for utilizing specialized sensors to gain novel insights into assessing smoking in real-life situations. To illustrate, a study explored the use of a wearable acoustic sensor designed to capture smoke-related sounds. The sensor, consisting of a microphone attached to the throat using an adhesive, was employed to record breath sounds. The authors further developed an algorithm capable of automatically distinguishing between smoking and nonsmoking breaths, offering a promising advancement in smoking detection [43].

**Table 5:** Studies proposing alternative strategies for the detection of smoking behavior.

Study	Country	Type of wearable	Application	Sensor modality	Accuracy	Data availability	Autonomous/integrated
Cheng et al [44], 2019	China	Surface acoustic wave (SAW) sensor	To develop a sensor to absorb ambient tobacco markers	Gas	—^a^	Researcher	Autonomous
Echebarria et al [43], 2017	United Kingdom	Adhesive microphone	To monitor the acoustic properties of smoking breaths	Breathing acoustic signals	Sensitivity: >70%; specificity: >90%	Researcher	Autonomous
Gurtner et al [45], 2018	New Zealand	Wearable camera	To monitor children’s exposure to smoking	Images	—	Researcher	Autonomous
Imtiaz et al [46], 2020	United States	Wearable camera–based sensor	To capture images of cigarette smoking episodes	Images	—	User and researcher	Integrated with a smartphone app
Qiao et al [47], 2019	China	Photoacoustic spectroscopy (PAS)-based carbon dioxide (CO_2_) sensor	To demonstrate the applicability of a PAS sensor	Gas	—	—	Autonomous
Rahman et al [48], 2022	Australia	Chemoresistive sensor	To demonstrate the effectiveness of a sensor for nicotine detection	Gas	—	User and researcher	Integrated with a smart electronic device

^a^Not applicable.

With recent advancements in computer vision, there is an opportunity to explore novel approaches using images and video analysis for the detection of smoking episodes. This emerging field offers promising instruments to complement existing methods. Thus, a wearable camera for capturing various aspects of cigarette smoking behaviors (ie, smoking actions, smoking environment, and social interactions) was developed and tested. In this regard, the authors determined that positioning the camera on the eyeglass temple yielded the best results in terms of capturing images related to cigarette smoking while minimizing image blurriness across different body locations [46]. In a similar vein, some authors evaluated the extent and nature of the exposure of children living in households with a smoker, using image data. In this study, children wore wearable cameras around their necks for 4 days, which automatically took pictures every 7 seconds. The wearable camera proposed appears to have high utility for studying health behaviors in private spaces, including smoking episodes both in private spaces (ie, home and cars) and outdoors [45]. Interestingly, in a separate study, a surface acoustic wave (SAW) sensor specifically was designed to detect cigarette smoke in real time by adsorbing ambient tobacco markers [44]. Finally, another study demonstrated the effectiveness of a highly sensitive photoacoustic spectroscopy–based CO_2_ trace gas sensor. This sensor holds the potential for detecting carbon dioxide (CO_2_) levels resulting from cigarette smoking, among other real-world applications [47]. These advancements in sensor technologies provide promising avenues for further research in the detection and analysis of smoking behaviors.

Another study proposed a chemoresistive sensor for real-time monitoring of nicotine vapor from e-cigarettes in the air. In particular, the authors proposed a vanadium dioxide (VO_2_)-based nicotine sensor integrated with an epidermal near-field communication (NFC) interface that enables battery-free operation and data transmission to smart electronic devices to record and store sensor data to detect nicotine at ambient concentrations [48].

## Discussion

### Principal Results and Comparison With Prior Work

The real-time monitoring of smoking behavior presents a significant challenge for public health research and the development of effective smoking-cessation interventions. However, advancements in mobile health (mHealth) technologies offer promising opportunities to address this challenge [13,16]. Through our scoping review, we have identified 37 studies published between 2012 and 2024 (March 10) that explore the use of wearable devices for assessing tobacco smoke exposure. These studies highlight the ongoing evolution of research in this field. Currently, research in the field predominantly focuses on the utilization of single or combined sensors to detect behaviors associated with smoking episodes. The inclusion of wearable sensors, particularly those integrated into elastic bands, has emerged as a noninvasive and mobile approach that enables continuous monitoring of smoking exposures. These advancements in sensor technology offer valuable insights for the development of targeted preventive measures and policies. In general, traditional smoking cessation programs, whether delivered in-person or via a smartphone, have proven efficacy but are constrained in their reach and utilization. In contrast, digital solutions exhibit greater potential for widespread accessibility, and there is evidence suggesting their efficacy. However, only a limited number of scientifically validated apps have been developed for commercial purposes, limiting their full potential for reach. Moreover, most digital solutions for smoking cessation have predominantly relied on a single form of technology, such as text messaging or an app. Mobile apps hold great promise in supporting patients in health care and promoting healthy behavioral changes. However, the success of these apps is largely determined by their features, influencing patients’ attitudes toward their use. In this context, the varied range of wearable sensors and studies highlighted in our scoping review have the potential to provide individuals with valuable approaches that fulfill both educational and motivational purposes. Furthermore, these wearable sensors can be smoothly integrated into evidence-based smoking cessation initiatives, thereby improving the overall effectiveness of such programs and encouraging a heightened interest in participating in cessation programs [49,50].

The studies included in our analysis demonstrate the efficacy of wearable sensors in monitoring smoking exposure. These sensors, deployed in various wearable bands, facilitate the detection of hand-to-mouth gestures, arm movements, and smoke inhalation during smoking episodes [12-14]. These sensors have also been employed to classify presmoking episodes [15] and evaluate temporal and emotional patterns associated with smoking behaviors [16]. Of particular interest is the use of a wearable forearm band, which allows for the monitoring of nicotine levels in sweat. This novel approach addresses the limitations of existing smoke monitoring devices and provides the potential capability to assess tobacco smoke exposures [11].

Overall, our findings highlight the ongoing challenges in achieving a personalized assessment of tobacco smoke for individuals in real-life settings. This suggests the need for further efforts to implement the proposed applications and enable a more precise evaluation of tobacco smoke exposure. It is crucial to consider personal factors, such as gender, age, and BMI, as well as contextual information including location, activity, and social context, in order to deeply characterize smoking patterns.

The focus of research in this field is shifting toward the utilization of complex systems comprising various types of sensors. For example, the integration of accelerometers and gyroscope sensors in a wearable smartwatch, along with wrist and chest bands, has demonstrated potential in detecting both active and passive tobacco smoking [17,18]. Some studies have explored noninvasive wearable devices that continuously collect real-time data on multiple physiological and environmental parameters, such as breathing patterns, chest movement, hand-to-mouth gestures, and lighting events. Among these innovative systems, the PACT system stands out, incorporating a comprehensive array of sensor modalities. This includes a chest module, a wrist or forearm band, an instrumented lighter, and a data logger. The findings from these studies have indicated that anthropometric measures of individuals can impact the signals collected by the PACT system. Additionally, they have suggested that breathing patterns show individual characteristics and are closely associated with hand-to-mouth gestures [28,29,31,32].

Several studies have introduced an advanced version of the PACT system, known as PACT 2.0, which offers several advantages and additional features. This upgraded system collects additional information, including GPS data, bioimpedance data, and heart activity, providing users with real-time feedback through a mobile app. Remarkably, the PACT 2.0 system has undergone development and validation to ensure its effectiveness in monitoring tobacco smoking in real-life situations, achieving a high level of acceptability [33]. Furthermore, it has been found that heart rate parameters can serve as an additional indicator of cigarette smoking, adding to the system’s capabilities [34]. The implementation of the PACT 2.0 system has yielded promising results among smokers, presenting opportunities for various applications in the field of public health research [35]. Notably, data collected through the PACT 2.0 system have revealed that hand-to-mouth gestures exhibit a high degree of regularity during smoking events. This finding suggests that the system can serve as a valuable tool for accurately identifying smoking episodes amidst various daily activities [36].

One notable advantage of these approaches is their ability to monitor without relying on self-reporting by smokers. This eliminates potential biases caused by underreporting. Moreover, the use of the PACT system only requires the cooperation of subjects in wearing the system, offering the potential to capture smoke exposure parameters that are not accessible through other methods. In addition, the information recorded by the PACT system can enhance our understanding of the health consequences associated with smoke exposure by evaluating the relation between biomarkers (eg, blood levels of CO_2_ and cotinine) and individual smoking behaviors. The PACT system also provides a unique advantage in its ability to capture comprehensive data on the complete breathing cycle during smoking, including puff, smoke inhalation, smoking apnea, and smoke exhalation. This is in contrast to traditional puff topography devices that only measure and analyze the air drawn through a cigarette during a puff. This comprehensive data can offer valuable insights into smoking behavior and its effects on health outcomes.

However, it is important to acknowledge some limitations of these systems. One such limitation is the relatively large size of the sensors, which are typically mounted on garments or vests. To overcome this limitation, there is a need to significantly miniaturize the sensors and integrate them into a fully wearable device, ensuring greater comfort and convenience for users. Overall, while these systems show promise in improving the monitoring of smoke exposure, it is essential to address the limitations by developing more compact and integrated sensor solutions for enhanced usability and effectiveness.

In this context, the AutoSense system emerges as a valuable combination that can capture a range of information, including heart rate, respiration, skin conductance, and physical activity. Its versatility opens up promising applications in various settings such as health care, workplaces, and communities. AutoSense not only enables the collection of physiological measurements but also offers particular potential in assessing stress response. The real-time transmission of data to a smartphone allows for the monitoring of physiological responses to real-life stressors and the continuous estimation of stress levels. This capability positions AutoSense as a valuable tool for evaluating behaviors associated with stress, such as drinking, smoking, physical activity, movement patterns, and conversations. Furthermore, it provides insights into physical, behavioral, and mental health conditions. Looking ahead, ongoing research in this field holds the promise of developing effective prevention and intervention strategies. The ability to deliver these strategies directly on smartphones aligns with the vision of mHealth, empowering individuals to proactively manage their health and well-being. By harnessing the potential of AutoSense, we can anticipate advancements that contribute to improved health outcomes and enhanced quality of life.

Some authors thoroughly discussed user behavior, comfort levels, and compliance with the use of wearable sensors in real-world settings [8,13,33,37,46]. To address this, brief acceptability questionnaires were administered to gauge users’ personal experiences with the wearable sensors. For instance, the results from these assessments indicated a strong inclination among participants to continue wearing the wearable camera for any subsequent multiday experiments [46]. This positive sentiment also encompassed other wearable accessories, such as elastic bands [13], and the PACT 2.0 system [33], highlighting that these devices were not only comfortable but also considered acceptable for prolonged use beyond the laboratory setting. Participants expressed contentment with the ease of wear for these technologies over extended periods.

As denoted in our work, there has been significant interest in sensors as a potential solution to address challenges related to the detection of tobacco smoke exposures. However, it is essential to highlight that no single wearable method has demonstrated 100% accuracy in detecting smoking events under all circumstances, with certain technologies that may be effective in specific environments and others that may not yield satisfactory results in the same context. To address this issue, it is evident that no single device currently offers a complete and accurate solution. Moreover, sensor responses are often influenced by ambient factors, such as motion and clothing. Although we described various single-sensor and multisensor approaches, our work denoted a lack of comparative analyses of their respective efficacies. To date, indeed, there has been no comprehensive survey or comparison study conducted on these approaches, elucidating the strengths and limitations of sensing technologies and their applicability in real-world settings. Furthermore, there has been limited evaluation of the underlying detection algorithms and their comparative accuracy.

In our research, only the study conducted by Imtiaz et al [46] addressed specific ethical considerations related to wearable camera research. They recommended that researchers involved in such studies actively protect the rights, privacy, dignity, and well-being of the individuals being studied. Emphasizing the importance of voluntary informed consent, research efforts should strive to ensure that participants willingly agree to take part. Additionally, research participants should be informed about the level of anonymity and confidentiality guaranteed during the publication and dissemination of findings, along with the potential for data reuse. To adhere to these ethical principles, participants throughout the study were instructed to remove the sensor system in situations where privacy was expected, such as restrooms, during activities causing discomfort (like sports and water-related events), and upon requests by individuals in their vicinity [46].

In general, wearable sensors described in our work are commonly characterized by their lightweight, mobile, and convenient design. However, as of today, there is a notable gap in the literature regarding a comprehensive analysis of their cost-effectiveness. While the advantages of wearables, such as ease of use and adaptability to various settings, are frequently acknowledged, there remains a critical need for research that delves into the economic aspects of implementing these technologies. Understanding the cost-effectiveness of wearable sensors is crucial for widespread adoption and integration into various domains, including health care and environmental monitoring.

Future studies could explore not only the initial investment and production costs but also the long-term economic benefits, considering factors such as durability, maintenance, and the potential impact on health outcomes or performance improvements. A thorough cost-effectiveness analysis would provide valuable insights for decision-makers, researchers, and industries looking to leverage wearable sensor technologies while considering the economic implications of their adoption.

### Limitations

Our findings indicate that wearable devices for detecting cigarette smoking and assessing smoke exposure are still in the early stage of development and require further advancements. Therefore, it is crucial to acknowledge certain limitations when considering the implications of our review. First, the included studies evaluated various behavioral and physiological characteristics, such as hand gestures, breathing, and lighting, using wearable sensors during smoking episodes. However, there is a need for future research to explore the integration of wearable chemical sensors for detecting smoking and measuring exposure to both traditional and electronic cigarette smoke.

Second, the included studies did not extensively explore how external environmental factors might impact the accuracy and reliability of these wearable sensors. A thorough investigation into how factors, such as ambient smoke, weather conditions, and other pollutants, could influence sensor readings would provide valuable insights. Third, it is important to note that our review primarily focused on wearable device development and validation, involving a limited number of participants, which included both smokers and nonsmokers. Fourth, the considerable heterogeneity between the studies precluded the possibility of conducting a meta-analytical approach, thereby hindering the ability to synthesize data, as well as to offer a quantitative perspective on the efficacy or cost-effectiveness of the wearable sensors explored. On the one hand, this represents a limitation of existing studies, but on the other hand, it represents an important perspective for future research.

Finally, the potential for biases in the selection of studies, which could impact the representativeness and generalizability of the obtained results, needs to be considered. Addressing these biases could enhance the overall robustness and reliability of our scoping review’s conclusions.

### Conclusions

Our scoping review highlights existing evidence about single-sensor or multisensor wearables in the context of tobacco smoke detection. While their potential is evident, further advancements and investigations are necessary to deeply evaluate their full potential and assist both individuals and health care professionals in addressing smoking-related health issues, as well as designing effective public health strategies.

Overall, wearable sensors may hold great promise in assisting individuals in their journey to quit smoking by offering real-time feedback on their exposures and smoking behaviors, empowering them to make informed decisions and take control of their habits. Through our scoping review, we have identified both the challenges and possibilities associated with wearable devices, which can serve as valuable guidance for future research endeavors. By addressing the identified pitfalls and exploring the potential avenues of improvement, wearable devices may become even more valuable tools in supporting smoking cessation efforts. In this scenario, ongoing research is crucial to refine and optimize these devices, ensuring their practicality and reliability in real-world settings.

Indeed, further research is needed to enhance the accuracy and usability of these devices, enabling researchers and health care professionals to gain a better understanding of the health implications related to smoking and to develop effective preventive interventions. In addition, future studies should examine factors such as comfort, adherence, and acceptability of wearable sensors. In fact, while many wearable sensors have been validated and tested in controlled experimental settings, there is a scarcity of research on their accuracy and applicability in real-life contexts.

## Appendix

Multimedia Appendix 1 PRISMA (Preferred Reporting Items for Systematic Reviews and Meta-Analyses) checkist.

## Abbreviations

ASPIRE: Automated Smoking Perception and Recording
CReSS: Clinical Research Support System
ECG: electrocardiogram
IMU: inertial measurement unit
mHealth: mobile health
PACT: Personal Automatic Cigarette Tracker
RF: radio frequency
RIP: respiratory inductance plethysmograph
RQ: research question
SVM: support vector machine
